# The transcriptional landscape of the deep-sea bacterium *Photobacterium profundum* in both a *toxR* mutant and its parental strain

**DOI:** 10.1186/1471-2164-13-567

**Published:** 2012-10-29

**Authors:** Stefano Campanaro, Fabio De Pascale, Andrea Telatin, Riccardo Schiavon, Douglas H Bartlett, Giorgio Valle

**Affiliations:** 1 Department of Biology and CRIBI Biotechnology Centre, University of Padua, Via Ugo Bassi 58/B, Padova, 35131, Italy; 2 Scripps Institution of Oceanography, UCSD, 9500 Gilman Drive, La Jolla, CA, 92093, USA

**Keywords:** High-pressure adaptation, Deep sea, *Extremophile*, Transcription, *Operon*, RNA-seq, UTR, *Vibrionaceae*, *Photobacterium profundum*, ToxR

## Abstract

**Background:**

The deep-sea bacterium *Photobacterium profundum* is an established model for studying high pressure adaptation. In this paper we analyse the parental strain DB110 and the *toxR* mutant TW30 by massively parallel cDNA sequencing (RNA-seq). ToxR is a transmembrane DNA-binding protein first discovered in *Vibrio cholerae*, where it regulates a considerable number of genes involved in environmental adaptation and virulence. In *P. profundum* the abundance and activity of this protein is influenced by hydrostatic pressure and its role is related to the regulation of genes in a pressure-dependent manner.

**Results:**

To better characterize the ToxR regulon, we compared the expression profiles of *wt* and *toxR* strains in response to pressure changes. Our results revealed a complex expression pattern with a group of 22 genes having expression profiles similar to OmpH that is an outer membrane protein transcribed in response to high hydrostatic pressure. Moreover, RNA-seq allowed a deep characterization of the transcriptional landscape that led to the identification of 460 putative small RNA genes and the detection of 298 protein-coding genes previously unknown. We were also able to perform a genome-wide prediction of operon structure, transcription start and termination sites, revealing an unexpected high number of genes (992) with large 5^′^-UTRs, long enough to harbour cis-regulatory RNA structures, suggesting a correlation between intergenic region size and UTR length.

**Conclusion:**

This work led to a better understanding of high-pressure response in *P. profundum*. Furthermore, the high-resolution RNA-seq analysis revealed several unexpected features about transcriptional landscape and general mechanisms of controlling bacterial gene expression.

## Background

Piezophiles are microorganisms adapted for optimal growth at hydrostatic pressure greater than 0.1 MPa (1 Atm=0.101 MPa) [1,2] and a large number of these organisms has been isolated from deep-sea. Since the ocean depths constitute the largest portion of the biosphere [3] this environment has a great importance both for the total microbial mass and for the number of species that are present.

In the ocean, vertical transport of microorganisms may be accomplished in regions of upwelling and downwelling as well as through association of bacteria with sedimenting particles or with animals [4]; for this reason deep-sea organisms may experience pressure differentials during their normal life. Since many biological processes and cell structures are susceptible to pressure changes [5], a counteracting action is expected to occur, most likely by controlling gene expression in a pressure-dependent way.

*P. profundum* SS9 is a piezophilic bacterium member of the *Vibrionaceae* family, isolated at a depth of 2500 m [6]; it has an optimal growth pressure of 28 MPa, but it is also able to grow at atmospheric pressure (0.1 MPa). This organism is only moderately piezophilic, thus offering some advantages over obligate piezophiles, for example it can be manipulated at atmospheric pressure and its response to pressure variations can be easily monitored. Furthermore, it is amenable of genetic manipulation and its genome has been completely sequenced [7]. One of the most interesting characteristics of this bacterium is its ability to modulate the abundance of several outer membrane proteins (OMPs) in response to pressure [8,9]*,* in particular OmpH and OmpL. OmpH increases its abundance 10–100 times as the hydrostatic pressure rises from 0.1 to 28 MPa, while OmpL decreases its abundance in the same pressure range [8-10]. These genes are under the control of ToxR, a transmembrane regulatory protein present in all *Vibrionaceae* so far sequenced. ToxR was first identified in *Vibrio cholerae* in which it regulates several virulence factors in response to changes in environmental parameters like osmolarity, pH and external amino acid abundance [11]. The ToxR regulon of *Vibrio cholerae* is very complex and, in conjunction with ToxS*,* TcpP*,* TcpH and ToxT*,* is involved in the regulation of a large number of genes [12]. Interestingly, the *toxR* orthologous gene in *P. profundum* SS9 is required for pressure-dependent regulation of OmpH and OmpL in response to hydrostatic pressure changes [10,13].

Numerous studies indicate that transcriptome reconstruction may be very useful to understand bacterial adaptation to different environments. Also small and compact genomes like that of *Helicobacter pylori* revealed an unexpectedly complex transcriptional landscape [14], while transcriptome reconstruction has helped in unravelling unclear phenotypes and in identifying complex sRNA regulatory networks [15]. In the cyanobacterium *Synechococcus elongatus* RNA-seq analysis led to the identification of significant Rho-independent transcription terminations resulting in interesting transcriptional structures [16].

In this paper we describe a study based on RNA-seq, performed in order to compare the transcriptional profile of the *P. profundum toxR* mutant (TW30) with the parental strain DB110 (a derivative of the wild type strain SS9). These two strains were grown at two different pressures (0.1 MPa and 28 Mpa) in order to perform a detailed analysis of pressure-regulated genes. The high-resolution potential offered by RNA-seq analysis allowed a complete characterization of bacterial transcriptional architecture at a level of detail that was previously not possible, highlighting many genes that are directly or indirectly regulated by ToxR. Moreover, the genomic annotation of this bacterium has now been considerably improved and includes many previously unidentified genes as well as a better characterization of the 5^′^ and 3^′^-UTRs and several hundred untranslated small RNA genes (sRNAs).

## Results and discussion

### The photobacterium profundum transcriptome

#### Global expression of chromosomes 1 and 2

We have analyzed the *P. profundum* transcriptome structure in DB110 strain and *toxR* mutant (TW30) both grown at 0.1 MPa (low/atmospheric pressure - LP) and 28 MPa (high pressure/280 Atm - HP). In Table 1 we have reported the number of reads aligned on the reference genome for each sample, only 18-35% of the reads have a single alignment. A large fraction of the remaining sequences were obtained from rRNA transcripts. Despite the sample obtained from DB110 strain grown at 0.1 MPa gave a high number of reads, the number of those having a single alignment is quite low; we hypothesize this could be due to a lower efficiency in rRNA depletion during the preparation of this sample. In fact the design of the capture oligos of the MICROBExpressTM kit (Ambion) do not take into account the *P. profundum* rRNA sequences and probably for this reason the rRNA depletion efficiency is not very high. In the first column of Table 1 are reported the strains (DB110 parental, TW30 *toxR* mutant) and the conditions (28 MPa and 0.1 MPa) analysed, in the second column the throughput obtained for each sample, in the third column the total number of reads aligned on the reference genome (*P. profundum* SS9 strain, assembly ASM19625v1) and on the fourth column only the reads having a single match on the *P. profundum* genome. In the fifth and sixth columns are reported the ratios between the number of reads obtained in the conditions analysed in comparison to the reads in the reference condition.

**Table 1:** Summary of SOLiD sequences produced for each sample

**Strains and conditions**	**Throughput (Mbp)**	**Absolute counts**		**Ratios**	
		**Reads aligned on reference genome**	**Uniquely aligned reads**	**Ratio considering DB110 HP as reference (all reads)**	**Ratio considering DB110 HP as reference (unique reads)**
DB110 28 MPa (DB110 HP)	582	16.621.219	5.836.186	1.00	1.00
DB110 0.1 MPa (DB110 LP)	584	16.690.818	3.050.616	1.00	0.52
TW30 28 MPa (TW30 HP)	611	17.466.471	5.692.495	1.05	0.97
TW30 0.1 MPa (TW30 LP)	479	13.699.146	4.517.864	0.824	0.77

The analysis of the 6441 genes (5683 from previous annotation and 758 identified in this study) showed that a large portion of the genome is expressed, in fact, depending on the condition examined, approximately 91-92% of the chr. 1 and 85-87% of the chr. 2 genes have at least two reads mapped (calculated from rough data, not normalized). Obviously this calculation is only a rough indication because it depends from the total number of reads and growth conditions examined. Despite this, our findings are not too different from those calculated for other bacteria with different methods. For example, Toledo-Arana and colleagues [15] analyzed *Lysteria monocytogenes* using tiling arrays and found that ∼ 98% of the genes are expressed.

**Figure 1:**
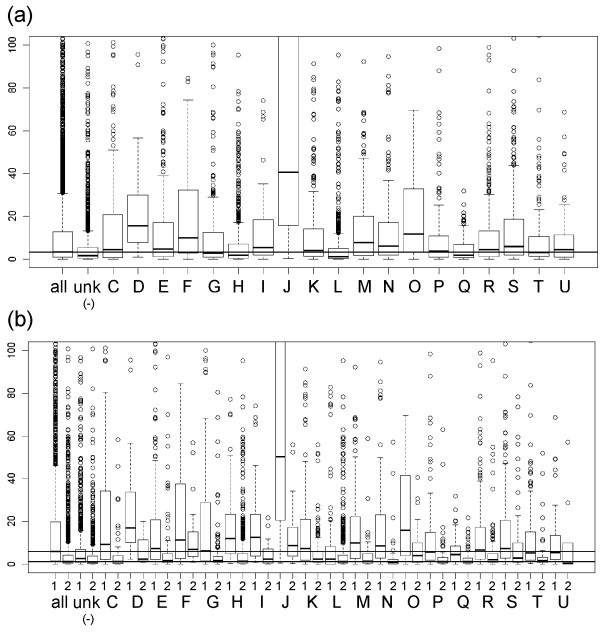
**Coverage of the genes belonging to different COG classes.** (**a**) Coverage distribution (mean values) of the genes belonging to each COG class in the DB110 (parental strain) at 28 MPa. Genes belonging to COG classes “J”, “D”, “O”, “M”, “F”, “S” have a significantly higher expression than the global gene expression value (p value < 0.0001), while COG classes “H”, “L”, and the genes coding proteins that do not belong to a COG class (unk) have a lower gene expression. Values determined for all the genes taken together are reported in the first column (all). We obtained similar results at 0.1 MPa and on TW30 strain (data not shown). (**b**) Comparison between the mean expression values on chr. 1 (1) and chr. 2 (2). It is evident that each COG class has on average a lower expression on chr. 2 (numeric values are reported on Additional file 1: Table S1). Horizontal lines indicate the median value for all the genes of the chrs 1 and 2.

Statistical analysis indicates that some COG classes “J” (Translation), “D” (Cell division), “O” (Posttranslational modification, protein turnover, chaperones), “M” (Cell envelope biogenesis, outer membrane), “F” (Nucleotide transport and metabolism), “S” (Function unknown) have an expression significantly higher compared to the global gene expression (p value < 0.0001), while classes “H” (Coenzyme metabolism ) and “L” (DNA replication, recombination and repair )and genes that are not assigned to a specific COG class (unk) have on average a lower expression (Figure 1A). Results are reported independently for chrs. 1 and 2 in Additional file 1: Table S1.These results are to a certain extent expected because class “J” contains genes coding for ribosomal proteins, “O” includes highly expressed chaperons like GroEL, DnaK, “D” includes a small number of very highly expressed proteins like FtsZ and FtsA. COG class “M” is of particular interest for deep-sea adaptation (see discussion below) because of the relevance of outer membrane proteins (for example porins and ABC transporters) to the uptake of nutrients present in low concentrations in the oligotrophic abyssal environment.

The percentage of genes having a medium/high expression on chr. 1 (mean coverage >= 10) is extremely high (26-38%) in comparison to the chr. 2 (7-14%), this confirmed the data previously obtained with microarrays [7] and was verified for all the strains and the conditions examined (Additional file 2: Figure S1).

In all *Vibrionaceae* examined, the distribution of COG classes on chr. 1 is very different from that of chr. 2 [17], for example in *P. profundum* COG classes (“J”, “D”, “O”, “H”, “F”, “M”) are overrepresented on chr. 1, while “G” (carbohydrate transport and metabolism) is overrepresented on chr. 2 [7]. COG classes containing highly expressed genes largely overlap those that have more representation on chr. 1. Since replication of bacterial chromosomes increases copy numbers of genes located near origins, highly expressed show a clear tendency to locate near the origin of replication [18]. Such differential gene dosages may be particularly important for vibrios as they harbour two chromosomes of different size [19,20] resulting in increased gene dosage differences within the larger chromosome relative to the smaller one. In fact the relative abundances of origin proximate DNA with respect to the terminus proximate DNA appear to be higher for the large *Vibrio* chromosomes than the small one [20]. This indicates that the higher gene expression on chr. 1 is mainly determined by the tendency of genes highly expressed (that belong to specific COG classes) to be localized near chr. 1 origin.

As suggested by Dryselius and colleagues [20] COG category “L” (Replication, recombination and repair) in *Vibrio cholerae*, *Vibrio vulnificus*, *Vibrio fischeri* and *Vibrio parahaemolyticus* is over-represented on the early replicated part of the large chromosome, and only *P. profundum* deviates from this pattern. *P. profundum* genome harbours a large number of transposable elements that belong to the “replication, recombination and repair” category, this can explain its peculiarity. As reported in Figure 1a, genes belonging to “L” group have a low expression in *P. profundum* and, despite the repetitive nature of these genes make them difficult to analyse using RNA-seq, this is a possible explanation for the different gene distribution in *P. profundum* with respect to other *Vibrio* species.

#### Operon structure

As described in the materials and methods section, alignment of the RNA-seq reads on the *P. profundum* genome allowed to identify transcripts boundaries and the *P. profundum* global operon structure.

This analysis was performed independently on the two strains and the two conditions examined in order to identify putative differences in operon structure. We identified a number of operons that are quite similar in structure regardless of the conditions and strains examined, ranging from 348 to 429 (Additional file 3: Table S2) and representing from 873 to 1072 genes (Additional file 4: Table S3). Since operon identification was performed only for genes having median coverage higher than two, variations in number of transcriptional units (TUs) among different growth conditions could be influenced by the number of sequences obtained for each sample. For this reason we normalized the data considering only the genes expressed. In this respect, the percentage of genes in operons range from 23.8% to 25.9% but this analysis emphasizes a strong difference between chr. 1 and chr. 2. In chr. 1 the percentage ranges from 30.6 to 32.9%, while in chr. 2 it is comprised between 7.7% and 10.9%, clearly showing that genes located on chr. 2 are “less transcriptionally organized” and are normally expressed as monocistronic transcripts. It is known that genes subjected to horizontal gene transfer (HGT) are not particularly prone to be in operons [21] but this can be only a minor determinant because HGT regions are only slightly overrepresented in the chr. 2 [22,23]. It is noteworthy that the percentage of genes transcribed as polycistronic RNAs (calculated with respect to the total number of genes having median coverage higher than 2) in chr. 1 is very close to that predicted in the archeon *Halobacterium salinarum* (32%) using tiling arrays [24] but completely different from those predicted using RNA-seq in *Helicobacter pylori* where 87.5% of the genes are expressed as polycistronic elements [14].

The number of genes belonging to the same operon ranges from 2 to 9 and the more abundant operons are those containing a lower number of genes (Additional file 5: Figure S2), this is a general trend previously reported in other bacteria [15].

The primary methods for operon identification in bacteria and Archaea are based on computational approaches that take into consideration intergenic gene distance and co-occurence in different species, as well as whether or not the genes participate in the same biological process [25,26]. Since our results are based also on gene expression, we have compared them with those obtained with computational methods, finding that 92.3-95.1% (depending on the strain and condition) of the gene couples predicted with our method are confirmed by the computational method of Price and colleagues [25]. The very close agreement between the two methods suggests that our approach is reliable.

Analyses of the polycistronic transcripts revealed a trend for genes belonging to certain COG classes to be organized in operons (Additional file 6: Table S4). We found statistically significant results for genes belonging to class “C” (energy production and conversion), “D” (cell division), “H” (coenzyme metabolism), “J” (translation), “M” (cell envelope biogenesis, outer membrane), “N” (cell motility and secretion), “O” (posttranslational modification, protein turnover, chaperones) and “U” (intracellular trafficking and secretion). Some of these results can be easily explained by the presence of some large operons such as those coding the ribosomal proteins (class J) (for example PBPRA0330-PBPRA0337), the NADH ubiquinone reductase (C) (PBPRA0823-PBPRA0828) and flagellar genes (N) (for example PBPRA0019- PBPRA0023). Since all these classes are overrepresented on chr. 1 [7] this can partially explain the higher number of operons identified on this chromosome.

#### Identification of small protein-coding genes

Analyses of the *P. profundum* transcriptional profiles indicate that intergenic regions (IRs) are frequently expressed. A comparative analysis among these putative new transcripts, the localization of the ORFs predicted using four different bioinformatics software (ORPHEUS, Glimmer2, Glimmer3 and GeneMark) [27-29] and the genomic position of the putative ribosome binding sites (RBS) (see materials and methods) lead to the identification of 298 “new” protein coding genes. Most of these genes were missed in previous annotation due to their small size (Additional file 7: Figure S3); in fact, their average length is 85 AA and during the gene finding process, ORFs shorter than 100 AA are frequently not considered as bona fide protein coding genes. ORFs identified using RNA-seq were further verified and annotated using BLAST search and COG (Additional file 8: Table S5). Notably, bioinformatics software cannot predict 58 of these small genes, but in 44 of these we were able to find a RBS in the correct position with respect to the putative start codon and 3 more have similarity with proteins identified in other bacteria. Among the 298 ORFs identified, 178 are hypothetical or conserved hypothetical proteins, while 120 have similarity with proteins having known function.

Some of the proteins that were missed in the previous annotation are of particular interest. For example the chi subunit of DNA polymerase III, the cell division ATP-binding protein FtsE, two tRNA synthetases, the heat shock protein HtpX, the outer membrane chaperone Skp (OmpH), and some components of the flagellum system.

Some of these proteins were identified as differentially expressed in the comparisons performed (see Additional file 9: Tables S11 and Additional file 10: Table S6 “all gene expression data” worksheet) providing *a first glimpse* of their *functional* properties. For example, 20 of these transcripts were found differentially expressed more than two times (p < 0.0001) in the experiment performed on parental strain grown at different pressures. In some cases we found a relevant expression change, for example the transcript coded by PBPRA0243a gene (a hypothetical protein) increases its abundance more than seven times increasing hydrostatic pressure from 0.1 to 28 MPa.

A comparative analysis performed considering RNA-seq coverage, the position of the putative RBS and the structure of the putative protein coding genes identified using the four software mentioned above, led to the identification of 144 genes with a wrong start codon prediction in our previous annotation (Additional file 11: Table S7). In 56 of these ORFs the TSS identified using RNA-seq was located downstream of the predicted start codon (Additional file 11: Table S7 column “K”, “confirmed by RNA-seq”) and a careful analysis of the RBS position allowed the identification of a new start codon that agree with the transcript structure. The 144 corrected ORF were translated in AA sequences and compared with the NCBI complete microbial genome database using a BLASTp search. Our predictions were confirmed by identifying orthologous genes in 134 cases (93%).

#### Massive number of small RNAs

The vast majority (460) of the new transcripts identified in *P. profundum* do not have the characteristics to be considered as *bona fide* protein coding regions. The main features that we have considered to classify transcripts as putative sRNAs are the absence of a RBS, low similarity with proteins identified in other bacteria and the absence of likely protein coding regions.

These putative sRNAs could be divided in different classes depending on their localization with respect to the flanking known protein-coding genes (Additional file 12: Table S8). The first class comprises 276 transcripts localized in IRs, while the second class consists of 69 transcripts that are partially overlapping with the 5^′^ or 3^′^-end of protein coding genes. 50 of the transcripts belonging to the second class overlap with the RBS of known protein-coding gene and this suggests that they could influence ribosome binding and thus possess a role in post-transcriptional regulation.

Analyses performed to define the coverage uniformity of the RNA-seq into known protein coding genes, indicates that a small fraction shows a marked coverage difference between the 5^′^ and the 3^′^ part of the gene. A careful manual analysis of these transcripts led to the identification of distinct regions having different coverage with respect to the rest of the ORF, that could represent cis-encoded sRNAs involved in determining the stability of the protein-coding transcript. We have classified these sRNAs in a third separate class comprising 115 putative transcripts (Additional file 12: Table S8).

Only a small fraction (29 on chr. 1 and 33 on chr. 2 out of 460) of these three classes of putative sRNAs have a similarity in Rfam database.

**Figure 2:**
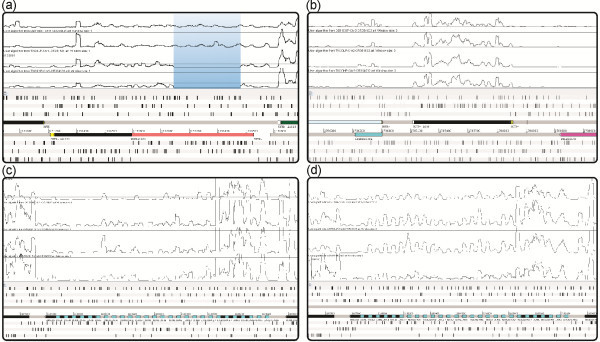
**Coverage profile of selected regions determined using RNA-seq.** Coverage refers to parental (DB110) strain at high (28 MPa, first lane from top) and low pressure (0.1 MPa second lane from top) and for the mutant (TW30) strain in the same conditions (third and fourth lanes). (**a**) *toxR* gene, the region having zero coverage in TW30 strain is due to the deletion on the gene. (**b**) Coverage determined using RNA-seq on the chr. 2 region containing the gene coding for the OmpC porin (an example of a differentially expressed gene). Note the very different coverage between the parental strain grown at high (first lane from top) and low pressure (second lane). The gene does not show difference in coverage comparing TW30 strain grown at high and low pressure (third and fourth lane). In the left part of the picture there is a feature representing a sRNA (PBPRB1638a) overlapped to the 3^′^-end of a protein-coding gene. A curious point is that, an analysis of the *E. coli* genome upstream of the gene coding the OmpC porin, revealed a sRNA in the same position. 5^′^ (white boxes) and 3^′^-UTRs (yellow boxes) of the genes are highlighted. (**c-d**) The chr. 2 region containing the CRISP elements gene cluster. In (**c**) we report the coverage determined by the reads uniquely mapped on the genome (maximum coverage is 25X), while in (**d**) the coverage is referred both to unique and repeated reads (maximum coverage is 50X). The blue rectangles are the Rfam predictions that clearly overlap with the RNA-seq coverage profile.

150 of the transcripts belonging to these three classes are localized in repeated regions. Despite the ambiguous assignment of these transcripts to a specific genomic region, some of these are of great interest. Similarity search performed in Rfam database indicates that a large region located on chr. 2 between the gene PBPRB1989 and PBPRB1991 contains a cluster of 27 putative CRISPR-DR4 elements (Figure 2 c-d). Moreover in the region located between PBPRA1278 and PBPRA1279 we found four tRNAs that were not identified using bioinformatic analysis.

It is widely known that trans-acting sRNAs localized in IRs have different roles with respect to the cis-encoded antisense sRNAs whose function remains largely unknown [30]. We have calculated the free energy of these different classes and of the 5^′^-UTRs using RNAfold software of the Vienna package [31] and we have compared our results with those obtained by random sequences having similar length and base composition. Our data confirmed that 5^′^-UTRs have higher free energy with respect to random genomic sequences and that this is mainly due to the low fraction of bases in secondary structures located close to the ribosome binding sites [16,32,33]. For this reason only 5^′^-UTRs longer than 150 bases tend to have a lower free energy with respect to random sequences (Additional file 13: Figure S4 a). Putative sRNAs localized in IRs tend to have a lower free energy (Additional file 13: Figure S4 b, c, d) and this probably reflects their different role and underlines the importance that secondary structures recover for the function of trans-acting sRNAs. The function of most of the putative cis-encoded antisense RNAs remains unknown, but Gene Ontology analysis of the corresponding protein-coding genes revealed that 2 GO classes are overrepresented: transcription with 16 genes (p-value 0.0014) and signalling processes with 13 genes (p-value 0.005) (Additional file 14: Table S9). This finding is of great interest since it has been demonstrated that two-components systems and sRNAs are interconnected in order to form networks that integrate and transduce information from the environment into fine-tuned changes of gene expression [34,35]. Although two-components systems usually act by modulating the expression of trans-acting sRNAs, it is also of great interest that putative cis-acting sRNAs are able to modulate expression of their cognate antisense genes.

In our experiment we found 5 genes out of 13 belonging to the signalling process are differentially expressed, two are slightly up-regulated in parental strain at high pressure (DB110HP vs. DB110LP); while three are down-regulated in mutant strain when compared to the parental at high pressure (TW30HP vs. DB110HP).

A very interesting point is that expression level of some putative sRNAs identified can change in response to hydrostatic pressure. Among the unknown sRNAs the more interesting are PBPRA0833b, PBPRA2131b, PBPRB0423a and PBPRA3306a, that are more expressed at 0.1 MPa (in comparison to 28 MPa) while PBPRA2336a, PBPRA2818a, PBPRA3027a and PBPRB0103a are more extensively expressed at high pressure (Additional file 10: Table S6). Among the sRNAs having similarity in the Rfam database, the putative untranslated 6S gene (PBPRA3120a) is more highly expressed at low pressure, while the putative untranslated RFN (PBPRB0898a) and the putative untranslated CsrB (PBPRB1150a and PBPRB1150b) are more highly expressed at high pressure. The RFN riboswitch [36] is located upstream of PBPRB0898 (3,4-dihydroxy-2-butanone 4-phosphate synthase) involved in the biosynthesis of riboflavin. The biologically active forms of thiamin (vitamin B1) and riboflavin (vitamin B2) are TPP and FMN/FAD, respectively. These cofactors participate in numerous metabolic pathways in all organisms. This could suggest a possible role of riboflavin in deep-sea adaptation.

#### *P. profundum* UTRs

RNA-seq allows the analysis of 5^′^and 3^′^-UTR regions. Using a self-written perl script the coverage of the regions upstream of the start codon and downstream of the stop codon were searched for a sharp signal reduction of the coverage value (SOM). Since the transcript structure of genes having a small number of sequences could not be reliably determined, this analysis was performed grouping together the reads of the four experiments and considering only the transcripts with an average coverage higher than 4. After manual verification of the data obtained and a comparison with predicted operons, we identified 2266 TSS (transcription start sites) and 2128 transcript ends (Additional file 15: Table S10).

Nearly 8% of the 5^′^-UTRs of the genes are shorter than 10 nucleotides and should be considered leaderless transcripts*.* This percentage is a little higher than that found in *H. pylori* where 2.2% of the protein coding transcripts are leaderless [14] but lower than that of *S. elongatus* that has 16% of leaderless transcripts.

**Figure 3:**
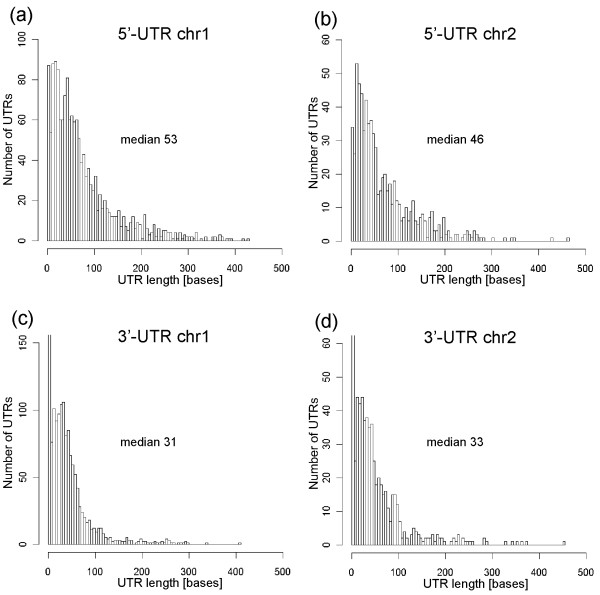
**Size distribution of the UTR regions in*P.profundum.*** 5^′^-UTRs on chr. 1 (**a**), and on chr. 2 (**b**), 3^′^-UTRs on chr. 1 (**c**) and on chr. 2 (**d**). On average 5^′^-UTRs are longer than 3^′^-UTRs, this is expected since regulatory information in bacteria is generally determined in 5^′^-UTR regions.

In general the size of *P. profundum* 5^′^-UTRs are very large in comparison with data obtained from other studies (median 51 bp) (Figure 3) [14-16,24,37,38] and there are not clear differences in size distribution between chr. 1 and chr. 2 (median 53 bp on chr. 1 and 46 bp on chr. 2). We have identified 992 transcripts with 5^′^-UTR longer than 60 nucleotides, since these could harbour novel cis-regulatory RNA structures [14]. There is a great potential for cis-regulation, at least if compared with the 337 long UTRs identified for example in *H. pylori*. Despite this, a similarity search performed in Rfam database using 1942 5^′^-UTRs longer than 20 bases leads to the identification of only 10 known RNA elements, suggesting that in *P. profundum* and probably in other bacteria the number of riboswitches with unknown function is very high. 

**Figure 4:**
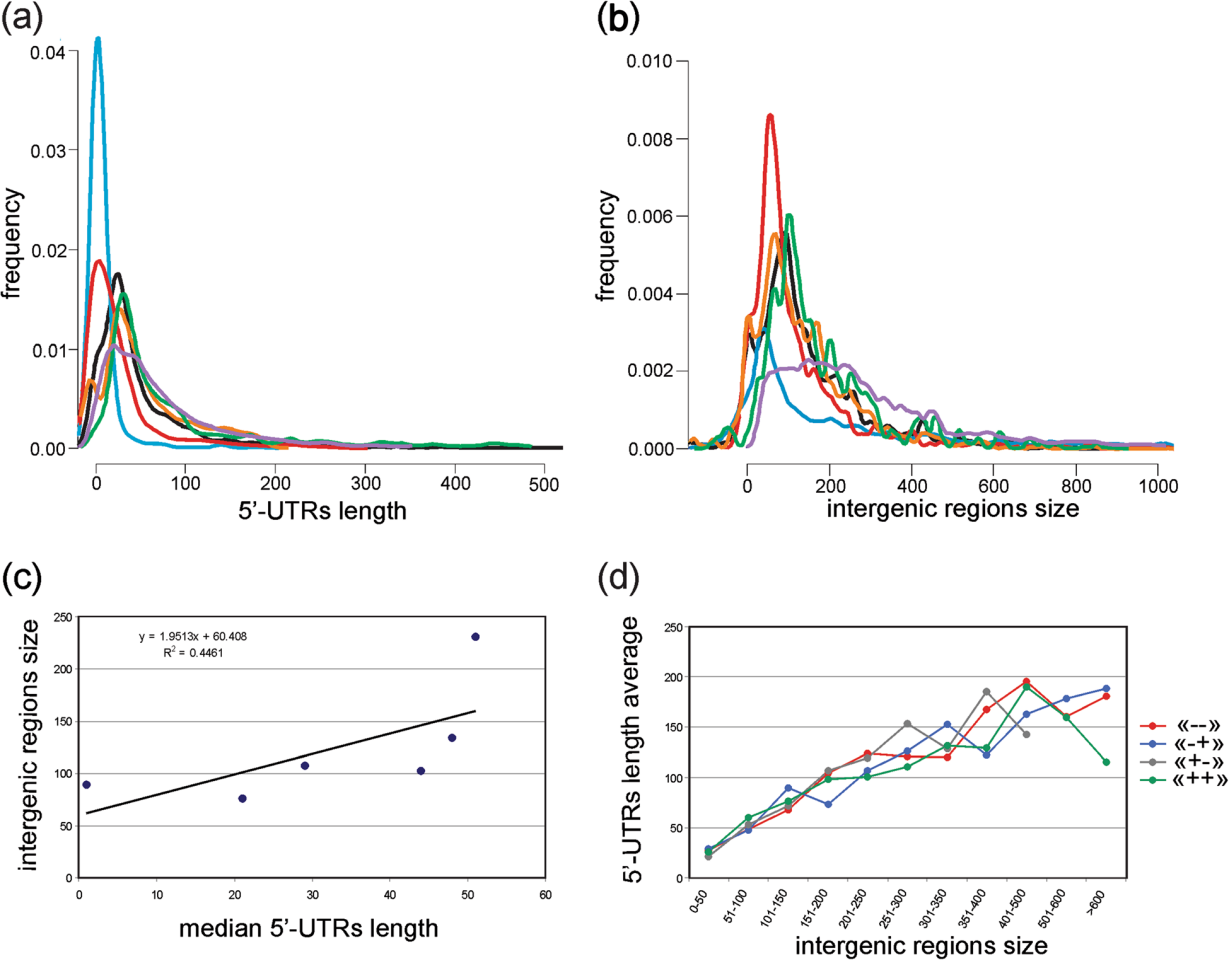
**Correlation between IRS and 5**^′^**-UTR length.** (**a**) distribution of the 5^′^-UTR length of different prokaryotes: *P. profundum* (purple), *Helicobacter pilory* (green), *Caulobacter crescentus* (orange)*, Synechococcus elongatus PCC 7942* (black)*, Halobacterium salinarum* (red) *and Sulfolobus solfataricus* (blue). (**b**) Distribution of the IRs length determined on the same six species. In (**b**) only IRs separating different operons were considered. (**c**) Median length of all the 5^′^-UTRs reported in (**a**) and of IRs reported in (**b**) is compared and interpolated. (**d**) UTR length is correlated to intergenic region size (IRS) size in *P. profundum*. IRs were classified in 11 classes according to their length and separated in four groups considering strand of the adjacent genes: double-regulatory regions, localized between two divergently transcribed genes (−+), single-regulatory (++) (− −) and non-regulatory (+−). “+” refers to forward strand and “–” to reverse strand. UTR length is correlated to IRS. Due to the very low number of (+−) IRs belonging to classes “501-600” and “> 600” these points were not plotted in (**d**).

We have compared the size distribution of the 5^′^-UTRs with those determined using tiling arrays and RNA-seq both in bacteria (*Caulobacter crescentus*, *Helicobacter pylori* and *Synechococcus elongatus* PCC 7942) and Archaea (*Solpholobus solphataricus* and *Halobacterium salinarum*) [14-16,24,37,38] (Figure 4a) and we have found that *P. profundum* have longer 5^′^-UTRs, particularly in the range 50–100 bp. 

IRs in bacteria are under selective pressure to minimize their size while maintaining the necessary regulatory sites. This is also supported by the observation that double-regulatory regions, localized between two divergently transcribed genes (DR or “-+” considering the coding strand of the flanking genes) are larger than single-regulatory (SR or “++” or “- -“) regions. It is also evident that different bacterial genomes have widely different average intergenic region sizes (IRS) and this is independent from genome size [33]. To explain this point we have recovered IRSs for the six prokaryotes considered in the analysis of the 5^′^-UTR size (Figure 4a). We followed the same procedure reported by Molina and colleagues [33] considering only IRs located between operons predicted using bioinformatics [25]. Comparison between median IRSs and median 5^′^-UTR size revealed that they are slightly correlated (R^2^ 0.45) (Figure 4c). This suggests that in bacteria (but this finding could also be extended to Archea) UTR region size is a relevant factor determining the great variability of the IRS observed between different species.

We performed an analysis considering only *P. profundum* IRs and UTR regions. We have classified IRs as suggested by Molina and colleagues [33] (“-+” DR; “++” SR; “--” SR; “+−” NR) and we grouped them in classes according to their size (0–50 bp; 51–100 bp, etc.). Figure 4d highlights that UTR size is roughly proportional (about 40%) to the length of IRSs. To verify this result we have selected all the 1330 IRs where both the UTRs of the flanking genes are known. All the IR classes (“++”; “- -”; “+−”; “-+”) gave similar results with UTR regions accounting for 38-43% of the IR. These findings support the hypothesis that large IRs have also a higher regulatory potential as described by Molina and colleagues [33], tending to harbour transcripts with larger UTRs that can have higher cis-regulatory potential.

**Figure 5:**
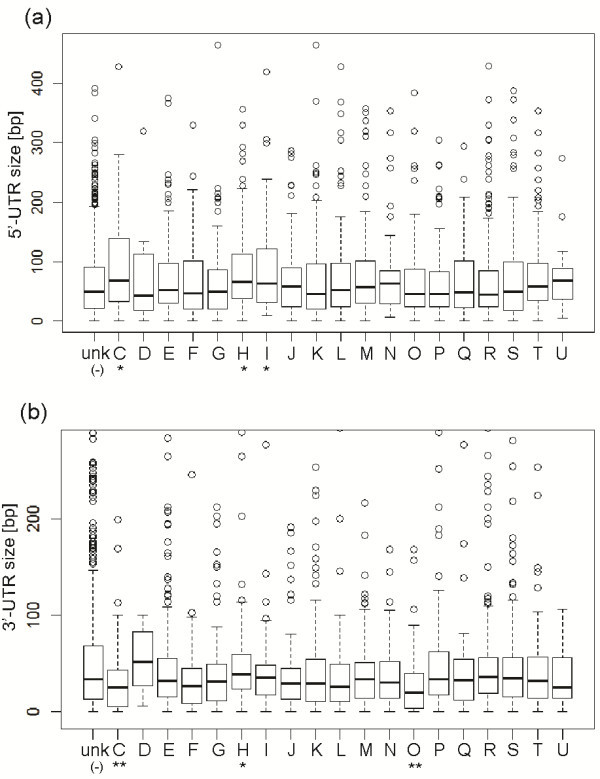
***P. profundum*UTRs size.** Size distribution of the 5^′^ (**a**) and 3^′^-UTRs (**b**) for different functional categories (COG). COG classes having significantly longer UTRs are marked using “*” symbol, those with significantly shorter UTRs are marked using “**”.

We have determined length distribution of UTRs for different functional categories (COG) and we detected significant correlations (Figure 5 and Additional file 15: Table S10). Genes belonging to the COG group “H” (coenzymes) have both longer 5^′^ and 3^′^-UTRs, those belonging to the “C” (energy) class have longer 5^′^-UTRs and shorter 3^′^-UTRs, those of the “I” (lipid transport and metabolism) group have longer 5^′^-UTRs and those of the “O” (protein modification) group have shorter 3^′^-UTRs. These results suggest that genes belonging to “H”, “C” and “I” groups require complex regulation. The “energy” group is of particular interest here because of its role in high-pressure adaptation (see next section). In general the 3^′^-UTR regions are shorter and contain a smaller amount of regulatory information in bacteria then the 5^′^-UTRs (median 32 bp *vs.* 51 bp in *P. profundum*).

Global analyses performed on chr. 1 and chr. 2 using RNAMotif [39] and RECOG (http://mbgd.nibb.ac.jp/RECOG/) indicated the presence of 3986 rho-independent terminators (0.7 per each protein-coding gene), while our RNA-seq analyses identified 2128 transcription terminator sites corresponding to 2317 protein-coding genes (in policistronic transcripts a single terminator site correspond to more genes) (0.92 per each protein-coding gene).

We were able to identify that 1153 transcription terminator sites (54%) corresponding to a specific rho-independent terminator, while 975 of the sites (46%) identified in our analyses do not have a correspondent rho-independent terminator predicted. 78% of the 1153 transcription terminator sites are within 50 bp of the terminator site, while 90.4% are within 75 bp (Additional file 16: Figure S5), this finding indicates that in general our prediction of the terminator sites agrees with those derived from bioinformatic analyses, but the size of the 3^′^-UTRs are slightly underestimated (Additional file 16: Figure S5). We can speculate that the presence of secondary structures in the terminator region could reduce the efficiency of retrotranscription resulting in a small underestimate of transcript length.

### The ToxR regulon and deep-sea adaptation

Analyses of the genes differentially expressed was performed considering only SOLiD reads mapping to one location in the *P. profundum* genome. This procedure prevented the analyses of repeated regions (like some parts of the genes coding for tRNAs and rRNAs or the CRISPR-DR4 elements) but it allowed a more reliable analysis of the remaining regions.

#### General discussion on differentially expressed genes

One of the aims of this procedure was the identification of genes differentially expressed in *P. profundum* cultures grown at low (0.1 MPa) (LP) versus high pressure (HP) (28 MPa) and to clarify the role of the ToxR transcription factor in high pressure adaptation.

In general, the impact of hydrostatic pressure variation on the parental strain transcriptome is quite strong, resulting in 2.9% of the genes (189 out of 6441) being expressed more than twice as much (p-value <= 0.0001) at high pressure (Additional file 9: Table S11), while 2.7% of the genes (174 out of 6441) are induced two-fold or higher at low pressure. The pressure effect is even stronger on the *toxR* mutant strain with 4.8% of the genes (303 out of 6360), the total number is lower because plasmid is absent in the mutant strain induced at high pressure and 2.9% (187 out of 6360) induced at low pressure. The major role played by the ToxR transcription factor in pressure-responsive gene expression was highlighted by the fact that the gene sets influenced by pressure changes in the DB110 and TW30 (*toxR* deletion) strains only partially overlapped (Additional file 17: Figure S6A and S6B, blue and yellow ovals, and Additional file 9: Table S11).

These general data bring out a clear alteration of the transcriptional profile in strain TW30, with a dramatic expression increase of numerous genes influenced by high hydrostatic pressure in strain DB110.

#### COG and GO analysis

Statistical analyses performed on COG and Gene Ontology of the differentially expressed genes gave a general overview of the biological processes involved.

The comparison performed between the parental strain at low and high pressure identified four COG categories statistically enriched (Additional file 18: Table S13): “C” (Energy production and conversion), “F” (Nucleotide transport and metabolism), “M” (Cell envelope biogenesis, outer membrane) and “P” (Inorganic ion transport and metabolism). As previously found [7] these results reflect the fundamental role of transport process in high pressure adaptation. Noteworthy, two of these four COG categories (“C” and “M”) were also identified in the comparison between parental and mutant strains highlighting the basic regulatory role of ToxR on gene classes involved in high pressure adaptation.

COG analyses were performed considering two gene expression thresholds (1.6 and 2-fold) and two p-values thresholds (0.001 and 0.0001) (see also Additional file 9: Table S11). Despite the more stringent threshold reduced the number of genes under analysis, we obtained very similar COG results (Additional file 18: Table S13).

Analyses performed considering Gene Ontology biological processes had substantially the same goal as the COG analyses but allowed the identification of more specific classes. To further refine the identification of specific classes involved in this mechanism, we performed different GO analyses for up and down regulated genes (Additional file 19: Table S12).

In the comparison performed between the parental strain grown at high and low pressure, we found 66 differentially expressed genes involved in transport of different compounds (for example monocarboxylic acid, chromate, cobalamin, glycolipids). Some of these processes were also statistically enriched in genes identified comparing the mutant and the parental strains and particularly in genes more expressed in the mutant strain at low pressure and in the parental at high pressure (Additional file 19: Table S12).

Genes located on chr. 2 and coding for ATP-synthase (PBPRB0130-PBPRB0137) were the main determinant of the GO categories and included “ATP synthesis coupled proton transport” and “energy coupled proton transport down electrochemical gradient” (see discussion below).

Finally one of the genes involved in GO class “response to superoxide” (PBPRB1551) belongs to a large gene cluster more expressed at high pressure in DB110 strain and localized on chr. 2. Although their expression is strongly decreased in the mutant strain (with respect to parental), they maintain their “pressure-dependent regulation” in mutant, indicating that these genes sense and respond to pressure using a ToxR-independent pathway. This gene cluster has been analyzed in detail in a previous work [7]. The genes of this cluster belong to the Stickland reaction pathway responsible for amino acid fermentation through an amino acid reductase containing selenocysteine. In this region we have also identified two small ORFs encoding proteins missed in previous annotation (PBPRB1548a-PBPRB1549a). Also these small ORFs are differentially expressed and both are annotated as “putative glycine reductase complex component A, selenocysteine-containing”. We have excluded the possibility that these small ORFs derived from sequencing errors by comparing their gene arrangement in both strains DB110 and 3TCK (that was sequenced and annotated independently) (Gordon and Betty Moore Foundation - http://www.moore.org/marine-micro.aspx).

#### Analysis of the ToxR regulon

The deletion in the TW30 strain [40] was clearly identified analyzing the distribution of the short reads on the *toxR* gene (PBPRA1022) (Figure 2). In the RNA-seq experiments performed on TW30 strain we did not find sequences aligned in the region located between 1132094 and 1132555 bp of the *toxR* gene (chr. 1) (from 563 bp to 102 bp downstream of the translation start site). This is a clear demonstration of the specificity of our RNA-seq approach. It was previously inferred that ToxR activity is regulated by a dimerization process that occurs at low-pressure, accompanied by an increase of protein level [13]. Our data indicates that there is also a control at the transcription level with a slight (1.4 fold) increase of the *toxR* expression level. The *toxS* gene, localized downstream of the the *toxR* gene in the same transcriptional unit, has a similar transcriptional behaviour.

**Figure 6:**
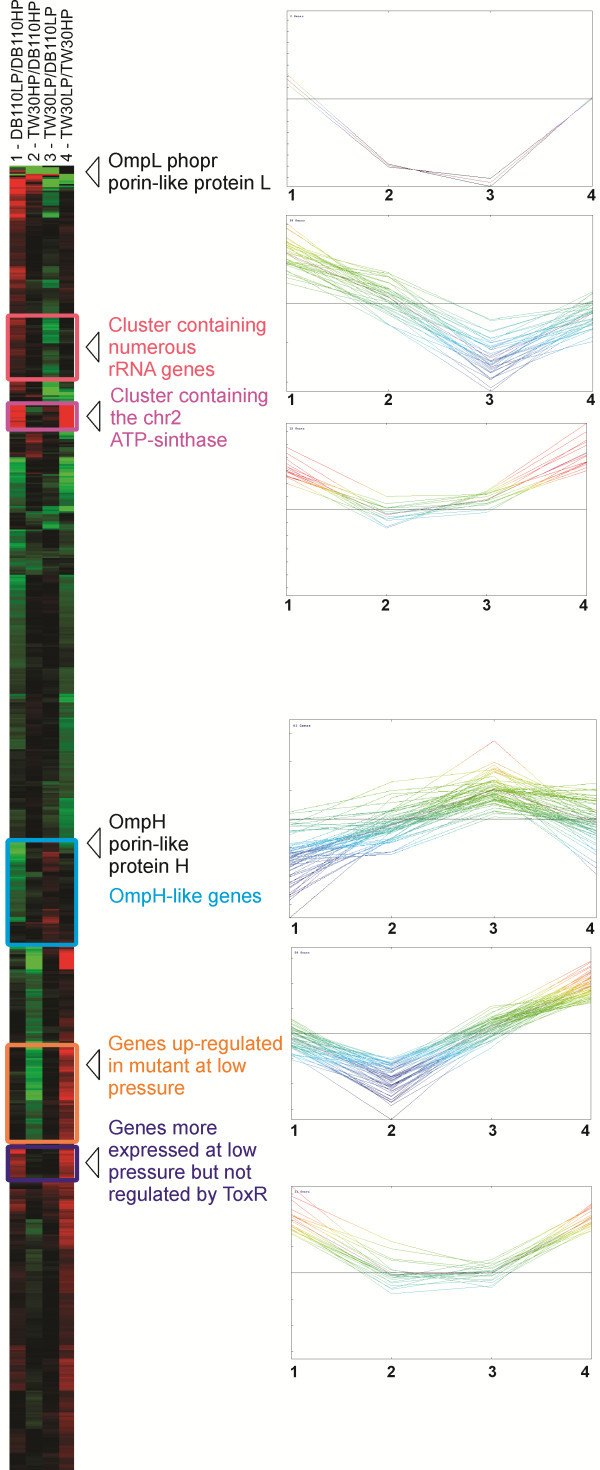
**Hierarchical clustering of the differentially expressed genes identified in the four comparisons.** The four columns of the cluster report in red and green the genes up and down-regulated in the four comparisons considered: (1) DB110 strain grown at low pressure *vs.* DB110 grown at high pressure (in red genes up-regulated at low pressure, in green those more expressed at high pressure); (2) TW30 strain grown at high pressure *vs.* DB110 strain grown at high pressure; (3) TW30 grown at low pressure *vs.* DB110 grown at low pressure; (4) TW30 grown at low pressure *vs.* TW30 grown at high pressure. Some gene clusters are highlighted in colour and a general description of the role of the genes is reported. The expression profile behaviour of the genes belonging to each cluster is visualized also in the right part of the figure using a different visualization.

As previously reported OmpH (PBPRA1012) and OmpL (PBPRA0600) porins expression is controlled by ToxR transcriptional regulator (PBPRA1022) [10,13,41]. RNA-seq experiments corroborate these results at transcriptional level confirming that ToxR is necessary for hydrostatic pressure regulation of these two genes and that *ompH* maintains its high expression level also in the mutant strain, while *ompL* expression is strongly reduced (Figure 6). 

Our data indicate that these two porins have a very high absolute expression level and this suggests their importance in membrane transport processes. *ompH* is the fourth most expressed protein-coding gene at high pressure in the parental strain (14889 RPKM) and *ompL* is the fourth most expressed at low pressure (12521 RPKM) *(*Additional file 10: Table S6); obviously we did not consider genes coding for rRNA transcripts because large parts of these transcripts have been removed before library construction (see materials and methods).

Another interesting point is that the number of genes having an “*ompH-like*” expression profile is larger than those having an “*ompL-like*” expression. This is visible from the Venn diagram reported in Additional file 17: Figure S6 but the global behaviour of the genes differentially expressed is also represented by the hierarchical clustering in Figure 6. In this clustering diagram we have highlighted the main gene clusters, reporting also the behaviour in the four comparisons in the small figure insets. From this image it is evident that the *ompL* gene is under the control of a fairly unique regulatory scheme, whereas many more genes exhibit “*ompH-like*” control (Figure 6). Results obtained from hierarchical clustering and euclidean distance calculation performed comparing each *P. profundum* transcript with *ompH* and *ompL* expression profiles indicates that 22 genes have an expression profile similar to *ompH* (Additional file 20: Table S14). Proteins encoded by these genes have very different biological functions and this may reflect the complexity of high pressure adaptation. For example PBPRA0126 encodes ferritin, a protein involved in iron storage. Various papers have recently demonstrated the competitive advantage conferred by the ability to cache iron, enabling certain bacterial species to dominate areas of the world’s oceans that are typically iron-poor, such as in the expanses around the Antarctic [42]. Interestingly large-scale iron fertilization might affect also deep-sea ecosystems as suggested by the microscopic pyrite particles emitted by the deep sea vents [43] and this indicates the importance of the iron in the deep sea. The ferritin expression control performed by ToxR could confer an important advantage to deep sea environments where iron is one of the limiting factors.

One of the more interesting genes identified encodes the OmpC porin protein (PBPRB1639) (Figure 2b). The orthologous gene in *E. coli* is necessarily required for hyperosmotic adaptation in the alkaline medium [44] and it was previously demonstrated that this gene is controlled by OmpR [45]. This led us to hypothesize that some similarities exists between the role of OmpR in *E. coli* and of ToxR in *P. profundum* and also between the osmotic stress response and high pressure adaptation. OmpR protein of *E. coli* has high similarity with various transcriptional regulators of *P. profundum*, in particular with OmpR-2 (PBPRB1908) that is strongly up-regulated at 28 MPa. OmpR-2 is differentially expressed both in parental and in mutant strains, this indicates that it can influence expression of genes in response to hydrostatic pressure independently from ToxR. This finding suggests that the transcriptional response to hydrostatic pressure is complex and, as previously explained, genes regulated by ToxR are only a fraction of the high number of genes influenced by pressure changes (Figure 6 and Additional file 17: Figure S6).

Finally we have compared the putative ToxR regulon determined in *V. cholerae*[12] and, curiously we found very small overlap with our results (Additional file 10: Table S6, comparison_with_Vchol_regulon). Out of 154 differentially expressed genes identified comparing N16961 strain and *toxR* mutant in *V. cholerae*, we were able to identify 117 orthologous genes in *P. profundum*. Analysis of results obtained in SS9 indicates that only 4 genes are differentially expressed in at least one of the two comparisons performed between DB110 and TW30. These genes are *ompL*, *nlpE* (an uncharacterized lipoprotein coded by PBPRB1923), the sigma-54 modulation protein coded by PBPRA3023 and the gene coding the outer membrane porin-like protein OmpT (PBPRA1128). In these two *Vibrionaceae*, ToxR has quite different roles, in fact in *V. cholerae* it coordinates expression of two critical sets of genes involved in bacterial virulence, while in *P. profundum* it regulates gene expression in response to hydrostatic pressure changes. The small number of regulated genes shared by these two microorganisms suggests that only those genes having a role in both of these two different adaptations (pathogenesis and deep-sea adaptation) are regulated by the ToxR transcription factor in both *V. cholerae* and in *P. profundum*.

#### Genes having expression profile different from ompH

The deep-sea is a very complex environment, characterized by a peculiar combination of chemical-physical parameters and nutrient sources. This raises the question whether organism adaptation to the abyssal environment requires complex gene regulation.

As previously mentioned clustering depicted in Figure 6 indicates that behaviour of differentially expressed genes identified in RNA-seq experiment is heterogeneous and could be grouped in various clusters. Besides the “*ompH*” or “*ompL*-like” gene clusters, the four other groups highlighted in Figure 6 are characterized by specific gene functions: the second cluster from top contains numerous rRNA genes, the third one includes genes localized on chr. 2 and coding for the F0-F1 ATP-synthase, the fifth cluster comprises genes involved in sulphate transport and utilization and the sixth cluster includes genes up-regulated at low pressure but not regulated by ToxR (for example the carbon-starvation protein A).

As previously mentioned, GO analysis reveals the presence of genes belonging to the “hydrogen transport” category (GO:0006818) (Additional file 19: Table S12). This result is due to the differential expression of the chr. 2 genes coding for F0-F1 ATP-synthase (PBPRB0130-PBPRB0137). Previous annotation indicates that this multiprotein complex is composed by eight subunits, while PBPRB0129 was annotated as a hypothetical protein. A BLASTp similarity search performed against “NR” and “Microbes” databases (http://www.ncbi.nlm.nih.gov/sutils/genom_table.cgi) found similarity only with hypothetical proteins. Despite this, both the results obtained from the transcriptome structure and also gene expression clustering indicates that PBPRB0129 is part of the “ATP-synthase gene cluster” (Figure 6). Using STRING database [46] we found that PBPRB0129 is part of the same multiprotein complex (both due to co-expression and co-occurrence) and finally, using the SMART database [47] we found that it has an ATP-synthase I domain.

Interestingly, “chr. 2 ATP-synthase” genes increase their expression when hydrostatic pressure decreases from 28 to 0.1 MPa, moreover at low pressure its expression is higher in TW30 strain respect to DB110 (Additional file 10: Table S6). In contrast, expression of the chr. 1 ATP-synthase (PBPRA3603-PBPRA3611) is not influenced by pressure, and its absolute expression level is very high (nearly 1505–2454 RPKM in parental strain at 28 MPa) with respect to the chr. 2 ATP synthase (58–138 RPKM in TW30 at 0.1MPa).

Similarity searches performed using BLASTp indicates that *P. profundum, Vibrio splendidus* and *Vibrio harveyi* have two different copies of the F0-F1 ATP synthase, one located on chr. 1 and one on chr. 2 while *Vibrio cholerae*, *Vibrio parahaemolyticus* and *Vibrio fischeri* have only one copy on chr. 1. Phylogenetic analysis performed using HOMOGENOM database [48] (http://pbil.univ-lyon1.fr/) clearly indicates that the gene clusters localized on chr. 1 are similar among all of these species and clearly distinct from those localized on chr. 2.

Taken all together these data indicate that the F0-F1 ATP synthase is duplicated only in a small number of *Vibrionaceae* species, the two copies are clearly different and possibly have evolved some specialized functions. Our gene expression data suggest that the two different complexes have a distinct role in *P. profundum* and are possibly related to high pressure adaptation. Pressure changes have a dramatic role in membrane organization and subsequently they have a strong effect on proteins involved in energy production and more generally on membrane proteins [9] (reviewed by Simonato and colleagues [49]). We can speculate that *P. profundum*, and possibly other *Vibrionaceae*, evolved the ability to control expression of the genes coding the “chr. 2 ATP synthase” to compensate reduction of functionality determined on the “main” ATP synthase by suboptimal environmental conditions. To our knowledge the other *Vibrio* species having two different ATP-synthase copies are not adapted to high pressure growth but *Vibrio harveyi* has been isolated both from shallow water and deep sea [50]. Further analysis will be performed in the future to understand if the two F0-F1 ATP synthase copies could be involved in high pressure adaptation in other *Vibrio* species.

In the *P. profundum* genome we have identified 11 porin genes. We have discussed above the role of three porins regulated by ToxR transcription factor OmpH (PBPRA1012), OmpL (PBPRA0600) and OmpC (PBPRB1639). Expression of two hypothetical maltoporin genes (codified by PBPRB2004 and PBPRB0413) is influenced by pressure but, curiously, the PBPRB2004 transcript is more abundant at 28 MPa in the parental strain, while the PBPRB0413 transcript is also more abundant at 28 MPa but only in the mutant strain. Moreover PBPRB0413 transcript displays a very high expression with respect to PBPRB2004. Finally, the gene PBPRA2139, belonging to the gram-negative porin superfamily has a higher expression at 0.1 MPa (with respect to 28 MPa) but only in the parental strain. This gene class is a clear example of the complexity of gene expression at different pressures as revealed by the comparison between parental and mutant strains at different hydrostatic pressures.

It has been suggested that OmpH provides a larger diffusion channel in the outer membrane respect to OmpL [51] and this could enhance growth and survival in nutrients-limiting environments like ocean depths, because of dissolved organic matter reduction with increasing depth [52,53]. Piezophiles could experience fluctuations in nutrient availability [54] and porins that are more expressed at high pressure (such as OmpH and maltoporins) could play the same role as the one suggested for *E. coli* OmpF in dilute nutrient environments. OmpF has a larger pore diameter with respect to OmpC allowing at least 10-fold higher diffusion rate than that provided by an equivalent amount of OmpC porin. This is particularly important in the case of large molecules, such as disaccharides [55]. This suggests that the ability to modulate porin abundance, especially those involved in the diffusion of large compounds, could be relevant in the survival strategy of the piezophilic bacteria.

## Conclusions

Our results highlight the potential of RNA-seq in transcriptome structure analysis, both for the identification of differentially expressed genes and for the reconstruction of the transcriptional landscape. This study made it possible to increase the known number of *P. profundum* protein-coding genes by more than 5% through the identification of small genes missing in our previous bioinformatic analyses. The transcriptome study also revealed a high number of sRNAs and indicated that their expression can be modified in response to hydrostatic pressure changes.

The identification of UTRs pointed out the high complexity of the *P. profundum* transcriptome. *P. profundum* harbors 992 genes having long 5^′^-UTRs; this indicates a high potential for novel cis-regulatory RNA structures, much higher that generally found in other prokaryotes. On the other hand, the application of RNA-seq analysis to more bacteria will probably change our general understanding of UTRs and their distribution.

The correlation between UTR length and IRS was determined by comparing *P. profundum* with other bacteria and Archea and suggests that prokaryotes with very large IRs could have an extremely high cis-regulatory potential.

We have expanded and strengthened the role of ToxR in high pressure adaptation. Data obtained from the comparison of parental and *toxR* mutant strains provided new insights into the ToxR regulon of *P. profundum*, while a comparison with *V. cholerae* revealed very little overlap between the toxR-regulons identified in these two organisms. These results indicate that, even within closely related bacteria, evolution can dramatically modify regulons in order to enable the adaptation to widely different environments. Since ToxR can sense different environmental parameters, *Vibrio* species can use this sensor/transcription factor to respond to different stimuli, but this requires concomitant deep modification of the regulated gene cluster.

Finally, our data suggest that while ToxR plays a major role in regulating genes involved in high pressure adaptation the expression of many genes can change in response to hydrostatic pressure independent of the presence of *toxR*; this suggests that other transcription factors, for example OmpR-2, may also have a role in deep-sea sensing and adaptation.

## Methods

### Bacterial strains and growth conditions

P. profundum DB110, a Lac^−^, rifampin-resistant derivative of wild-type P. profundum SS9 [9], served as the parental strain for mutagenesis experiments.

TW30 strain is a *toxR* deletion mutant constructed by marker exchange-eviction mutagenesis [40].

Bacterial cultures were growth at 0.1 MPa and 28 MPa in Difco Marine 2216 Broth as previously described [7].

### RNA extraction, rRNA subtraction and cDNA synthesis

For RNA-seq analysis bacterial cultures were grown as explained above at two different conditions (0.1 MPa; 16°C and 28 MPa; 16°C); for each growth condition total RNA was extracted from three independent cultures using trizol (Gibco) and RNeasy columns (Qiagen). Genomic DNA was removed using DNAsi (Ambion). RNA quality was determined using the 2100 Bioanalyzer (Agilent). Equal RNA quantities obtained from the three cultures grown in the same condition were pooled to reduce biological differences between replicates and to obtain a more robust sample and representative of each condition examined.

Starting from 10 μgs of total RNA, 16S and 23S rRNAs were removed using the MICROB*Express*™ kit (Ambion), after rRNA depletion, RNA was fragmented incubating the sample at 82°C for 6 mins in a fragmentation buffer (40 mM Tris Acetate pH 8.1, 100mM KOAc, 30 mM MgOAc-4H_2_0). Fragments obtained were checked by gel electrophoresis, had an average length of 200 bases and range in quantity from 1 to 2 μgs (estimated using *NanoDrop* 1000 Spectrophotometer - Thermo Scientific).

All the RNA obtained from each sample was retrotranscribed using One-Cycle cDNA Kit (Invitrogen), second strand synthesis was performed using the same kit. Protocol was modified because first strand synthesis was performed using random hexamers. DNA obtained was purified using AMPure^R^ kit (Agencourt) and quantified using NanoDrop 3300 Fluorospectrometer (Thermo Scientific).

### Preparation of libraries and high throughput sequencing

Libraries were prepared following the SOLiD™ System 2.0 User Guide and SOLiD™ 2 System Template Bead Preparation guide and sequenced using the SOLiD V2 system (Applied Biosystems). To preserve abundance of the transcript in the final library, we used only eight amplification cycles after adaptor ligation. Details about the number of sequences obtained for each sample are reported in Table 1.

### Reads alignment

Reads obtained were aligned using PASS software [56]. Gene expression comparison between different conditions and strains was performed considering only reads uniquely mapped. Transcriptome reconstruction was performed using uniquely mapped reads or, alternatively, all the reads (unique and repeated) in order to improve the identification of the transcripts that are duplicated in the *P. profundum* genome like for example the CRISP elements and to better define the transcript structure of the genes having repeated regions. Moreover, since there are numerous features that are partially or completely overlapped, when possible we excluded from coverage calculation these regions and we considered only the portions that are specific for a certain gene. Moreover, despite the total number of reads mapped in the four experiments is quite similar (Table 1), to simplify the comparison between absolute expression values of the genes in different conditions and strains, we have normalized the absolute number of reads with respect to the parental strain at high pressure and, if not indicated, we refer to this normalized number (Additional file 10: Table S6).

Starting from PASS output and using self written PERL scripts we have determined a single base coverage files for chr. 1, chr. 2 and plasmid for each of the four experimental conditions analyzed. These files was visualized using Artemis browser [57] loading the files as “user plots” and this process was extremely useful for transcripts identification and manual verification of the results obtained.

### Analysis of sRNAs and protein coding genes

To identify the IRs that are transcribed, the coverage plot was manually checked and compared with bioinformatic analysis of the putative ORFs and with the putative ribosome binding sites (RBSs). ORFs prediction performed in our previous *P. profundum* SS9 annotation [7] was performed using ORPHEUS and Glimmer 2 [27,28]. In this project gene finding was performed again using Glimmer 3 and Genemark HMM4 [28,29] to improve small ORFs identification. Since this analysis is quite challenging we have chosen to identify all the putative RBSs of the *P. profundum* genome both to reinforce the gene prediction and to better discriminate sRNAs from small ORFs. RBSs were identified recovering the region 20 bp upstream of all the ORFs and analysing these regions using MotifSampler [58] to generate a weight matrix using a 6 bp “word”. Motif Sampler used a background model generated for the entire *P. profundum* genome using the CreateBackgroundModel software. The weight matrix was then used to identify all the putative RBSs in the genome using MotifLocator [58]. Finally we have considered all these data together to identify transcripts encoding small ORFs; all these evidences are reported in Additional file 8: Table S5. The putative small ORFs identified were then aligned using BLASTp in the NCBI microbial genomes database (http://www.ncbi.nlm.nih.gov/sutils/genom_table.cgi). For each protein the COG class was determined using the COGnitor software to compare our data with the Clusters of Orthologous Groups of proteins (COGs) database [59] (http://www.ncbi.nlm.nih.gov/COG/).

All the transcribed IRs lacking predicted small ORFs, were considered as sRNAs. Some putative small RNAs were also identified in regions that are overlapped to known protein coding genes, these were determined analyzing the coverage in different regions of each ORF: those having very different coverage (more than two fold) between 5^′^ and 3^′^ regions were manually checked to identify distinct regions having higher coverage.

Putative sRNAs identified were aligned in the Rfam database [60] (http://rfam.sanger.ac.uk/), results are reported in Additional file 12: Table S8.

### Identification of 5^'^ and 3^'^-UTRs

Identification of UTR regions have been performed using self-written PERL scripts. Starting from the translation start site and the stop codon, the script analyzed the coverage upstream and downstream the ORF in order to identify a point of very rapid coverage reduction. Reduction was identified calculating the average coverage for each gene (considering the protein coding region), the transcription start and termination sites were positioned exactly in the position where the coverage drop down to 1/20^th^ of the mean coverage value of the gene. This procedure was determined empirically. We did not choose to move upstream and downstream the coding region up to regions of zero coverage because this procedure, frequently used in programs for transcripts reconstruction, tends to overestimate the UTR size considering also regions having 1 or 2 fold coverage that are common upstream the highly expressed genes. Analysis was performed only for genes having a mean coverage equal or higher than 4. Finally, UTR regions identified were manually checked.

As discussed in results and discussion, data obtained for 3^′^-UTRs were also compared with the position of Rho-independent transcriptional terminators predicted using TransTerm [61] and RNA motif software [39].

### Operons identification

Operons were identified using a simple procedure previously developed for low density tiling arrays [62] and modified to manage RNA-seq data. Briefly, two adjacent genes located on the same strand was considered on the same operon if their coverage difference was lower that a specified threshold. This threshold was determined calculating the coverage variability between different regions of the same gene. For each condition examined we have calculated the log_2_ ratio between the mean coverage of the 5^′^-half of the gene and the 3^′^-half of the same gene and we have determined the standard deviation of the distribution of these values. We used a PERL script to estimate if the log_2_ ratio of the coverage of two adjacent genes located on the same strand was lower than one standard deviation. Finally we have improved the prediction considering the position of the 5^′^ and 3^′^-UTRs previously determined if available. We did not used directly the data determined for 5^′^- and 3^′^-UTRs for operon prediction because these data are more difficult to determined, they require a high coverage and were available only for a lower number of genes. At the end of the process these data were manually verified.

### Differentially expressed genes

Starting from PASS output files obtained aligning reads against the *P. profundum* genome and using self written PERL scripts, the number of reads uniquely mapped on each gene was determined (for protein-coding genes we considered only short reads mapped on the ORF region). This number was used to generate input files for DEGseq software [63] and determine differentially expressed genes. We have compared the genes identified as differentially expressed at different pressures in the wild type (DB110) strain (DB110 HP vs. DB110 LP experiment) and we have compared them with those identified in the mutant (TW30) strain grown in the same conditions (0.1 and 28 MPa) (TW30 LP *vs.* TW30 HP experiment). To better define the genes regulated by the ToxR transcriptional regulator, we have also compared gene expression between mutant strain (TW30) *versus* parental strain (DB110) at low (TW30 LP *vs.* DB110 LP experiment) and also at high pressure (TW30 HP *vs.* DB110 HP experiment) (Additional file 10: Table S6).

For each comparison between environmental conditions and strains we have classified the genes differentially expressed in four categories considering the degree of the differential expression (higher than two times or comprised between 1.6 and 2 times) and the p-value (lower or equal than 0.0001 and comprised between 0.0001 and 0.001) (Additional file 9: Table S11). Since the number of differentially expressed genes is high we have focused our attention on genes that are more differentially expressed (higher than two times) and having a low p-value (lower than 0.0001), if not differently specified in the discussion we refer to this gene class.

Cluster analysis was performed on all the differentially expressed genes identified using DEGseq software using MeV software [64]. Hierarchical clustering was performed using average linkage method and Euclidean distance calculation.

### COG, GO

Differentially expressed genes were analysed using GoMiner [65] in order to find GO classes statistically enriched with differentially expressed genes.

The COG categories were statistically evaluated to identify whether particular categories were overrepresented relative to genes in the whole genome. Calculations were performed using a hypergeometric distribution. The probability P of finding at least k genes of a functional category within a group of n genes was given by the following equation:

(1) $$P=\;\sum_{i=k}^{n}\frac{\left(\begin{array}{l} f\\ i \end{array}\right)\;\left(\begin{array}{l} g-f\\ n-\;i \end{array}\right)}{\left(\begin{array}{l} g\\ n \end{array}\right)}$$

where f is the total number of genes for each COG category and g is the total number of genes bound by MbRpoE/F within each specific COG category. Hypergeometric distribution was calculated using R statistical package (R Development Core Team, 2006) with a significance threshold of 0.05. The same software was also used to generate histogram plots.

Ortologous genes between *P. profundum* SS9 strain and *V. cholerae* were identified using RECOG software (http://mbgd.nibb.ac.jp/RECOG/). Parameters were set as “default” except that the best hit criterion used was “bidirectional”.

### **Submission of RNA-seq reads to public databases**

RNA-seq reads and tables with differentially expressed genes have been submitted to GEO database; entries have been approved, GEO accession numbers is GSE38259.

## Competing interests

The authors declare that they have no competing interests.

## Authors’ contributions

DHB, GV and SC conceived the work; DB grew the bacteria at high pressure and extracted RNA; RS, SC and FDF constructed and sequenced libraries; SC, FD and AT aligned sequences and performed identification of small RNAs and small protein-coding genes; SC annotated genes, performed analysis of UTRs, intergenic regions and statistical analyses; SC, DHB interpreted the data; SC drafted the manuscript, DB, GV revised the manuscript. All authors have read and approved the manuscript.

## Supplementary Material

Additional file 1 **Table S1. **Gene coverage calculated from SOLiD sequences uniquely aligned on the *P.profundum* DB110 strain at 28 MPa. Results obtained were clustered accordingly to COG classes and considered separately for chr. 1 (table cells numbers "1" at line 2) and chr. 2 (table cells numbered"2" at line 2). Lines 2–8 reported respectively: average of the coverage values calculated for genes belonging to each COG class, the minimum value, the first quartile, the median, the third quartile, the maximum value and the p-value (calculated using the Wilkoxon test). Data were graphically represented in Additional file 7: Figure S3. (XLS 38 kb) Click here for file

Additional file 2 **Figure S1. **Pie charts reporting the coverage of the *P. profundum* SS9 genes. (A-D) coverage of the chr. 1 genes, (E-H) coverage of the chr. 2 genes. Coverage was calculated at a single base level on the genome considering the uniquely aligned SOLiD reads and then converted to the mean coverage value for each gene. Click here for file

Additional file 3 **Table S2. **Number of operons identified in DB110 and TW30 strains at 28 and 0.1 MPa. In cells D4-G7 were reported the number of operons identified in the strains and the hydrostatic pressures analyzed; in cells I4-L7 were reported the number of genes belonging to the operons identified. Since chr. 1 and chr. 2 have different number of genes, we also reported the percentages of operons identified (cells N4-Q7). Operons identification was restricted to the genes having coverage higher than 2, for this reason in the calculation of percentage values we refer only to those having coverage higher than 2. Click here for file

Additional file 4 **Table S3. **Operons identified in the chrs 1, 2 and in the plasmid of *P. profundum*. Each worksheet refers to a specific experimental condition and strain. On each worksheet column (A) reports the number of genes belonging to each transcriptional unit, column (B) refers to the number of transcripts composed by a certain gene number. Column (C) and following report the genes belonging to each transcriptional unit. Click here for file

Additional file 5 Figure S2. Number of genes transcribed in operons of different lengths in DB110 and TW30 strains at low and high pressure. From the histogram it is clearly evident that operons composed by small number of genes are more abundant. This is a general trend in Bacteria. Data were reported for chr. 1 (azure-blue) and chr. 2 (red-orange) and the result is similar. chr. 2 has on average a lower percentage of genes organized in operons but in this graph the result is also due to the absolute gene number that is lower on chr. 2 than on chr. 1. Click here for file

Additional file 6 **Table S4. **Statistical analysis of the COG classes enriched in policistronic transcripts. Operons identified in all the four transcriptional analyses were considered in columns (C) (number of genes belonging to each COG class) and (D) (p-value), while those identified only in three, two or one experiment are reported respectively in columns (G-H), (I-J), (K-L). Graph reported below refers to the percentage of genes that are organized in operons in all the experiments considered (column E), results were calculated relatively to the total number of genes per class (column C). Description of COG classes is reported in column (M). Classes having p-values lower than 0.5% were reported in bold in table and in red in the graph. Click here for file

Additional file 7 **Figure S3. **Size distribution of protein coding genes. Histogram showing the size distribution in aminoacids (AA) of the protein coding genes identified in this experiment using RNA-seq data (B) *(*Additional file 6: *Table S4)* along with the protein coding genes identified in the *P. profundum* genome sequencing project (A) using bioinformatics. AA size is reported on the y axes and was limited to 2000 despite a very small number of protein coding genes has higher values. Click here for file

Additional file 8 **Table S5. **Main characteristics of the putative small ORF identified from RNA-seq experiment. Column (B): locus tag; column (C): localization on chr. 1 (1), chr. 2 (2) or plasmid; column (D): position; column (E): predicted protein size in AAs; column (F): strand; column (G): ribosome binding site predicted using MotifScanner (see materials and methods) is reported in red color and separated from start codon (in red) from black colored bases; column (H): RBS score calculated using MotifScanner software; column (I): BLASTp results obtained considering complete microbial genomes database (NCBI); column (J): gene name based on BLASTp similarity search; column (K): gene description based on BLASTp search versus complete microbial genomes and NR database; column (L): software (if any) confirming the ORF identified using RNA-seq; columns (M-N-O): COG prediction performed using COGnitior software (http://www.ncbi.nlm.nih.gov/COG/old/xognitor.html). (XLS 140 kb) Click here for file

Additional file 9 **Table S11. **Number of the genes differentially expressed identified in the four comparisons performed. In rows 2–14 are reported the data obtained for the comparison between parental strain grown at low (0.1 MPa) and high (28 MPa) pressure (highlighted in blue “DB110LP/DB110HP”). Genes that are expressed equal or more than two times at low pressure and having a p-value lower than 0.0001 are reported in cell (B3); genes expressed equal or more than two times at low pressure and having a p-value comprised between 0.001 and 0.0001 are reported in cell (B9); genes expressed between 1.6 times and two times at low pressure and having a p-value lower than 0.0001 are reported in cell (D3); genes expressed between 1.6 times and two times at low pressure and having a p-value comprised between 0.001 and 0.0001 are reported in cell (D9); genes expressed equal or more than two times at high pressure and having a p-value lower than 0.0001 are reported in cell (G3); genes expressed equal or more than two times at high pressure and having a p-value comprised between 0.001 and 0.0001 are reported in cell (G9); genes expressed between 1.6 times and two times at 28 MPa and have a p-value lower than 0.0001 are reported in cell I3; genes expressed between 1.6 times and 2 times at high pressure and having a p-value comprised between 0.001 and 0.0001 are reported in cell (I9); in rows 16–28 are reported the data for the comparison between mutant strain (TW30) and parental strain (DB110) at high pressure (28 MPa) (highlighted in violet “TW30HP *vs.* DB110HP”). in rows 30–42 are reported the data for the comparison between mutant strain (TW30) and parental strain (DB110) at low pressure (0.1 MPa) (highlighted in pink “TW30LP *vs.* DB110LP”). in rows 44–56 are reported the data for the comparison between mutant strain (TW30) grown at low (0.1 MPa) and high (28 MPa) pressure (highlighted in azure “TW30LP *vs.* TW30HP”). Click here for file

Additional file 10 **Table S6. **Gene expression analyses of the *P. profundum* SS9 genes. Column (A): starting from origin of duplication, genes are numbered according to the position on the genome and on chromosomes; column (B): locus tag; column (C): Swiss-prot ID; column (D): gene name; column (E): gene description downloaded from NCBI database; column (F): gene description obtained from CMR (Comprehensive Microbial Resource) (http://cmr.jcvi.org/tigr-scripts/CMR/CmrHomePage.cgi); columns (G-H: COG code and COD ID; columns (I, J, K, L): chromosome, strand, start and stop position of the genes; columns (M, N, O, P): number of reads mapped on genes in the four experiments (DB110 at 28 MPa, DB110 at 0.1 MPa, TW30 at 28 MPa and TW30 at 0.1 MPa. All values are normalized considering the total number of reads mapped on the reference sample (DB110 at 28 MPa); columns (Q, R, S, T): reads per kb per 1 million reads mapped (RPKM) values are reported for the same four samples (DB110 at 28 MPa, DB110 at 0.1 MPa, TW30 at 28 MPa, TW30 at 0.1 MPa); columns (U, V): log_2_ ratios and p-values calculated using DEGseq software for the comparison between DB110 0.1 MPa and DB110 28 MPa; columns (W, X): log_2_ ratios and p-values calculated using DEGseq software for the comparison between TW30 28 MPa and DB110 28 MPa; columns (Y, Z): log_2_ ratios and p-values calculated using DEGseq software for the comparison between TW30 0.1 MPa and DB110 0.1 MPa; columns (AA, AB): log_2_ ratios and p-values calculated using DEGseq software for the comparison between TW30 0.1 MPa and TW30 28 MPa; columns (AC, AD): log_2_ ratios and p-values calculated using DEGseq software for the comparison between TW30 0.1 MPa and DB110 28 MPa; column (AE): gene ID of the orthologous *V. cholerae* genes belonging to the ToxR regulon; column (AF): gene name; column (AG): gene function; column (AH): gene description; column (AI): gene ID; column (AJ): log_2_ ratio of gene expression values determined in *V. cholerae toxRS* mutant compared to N16961 strain; column (AK): log_2_ ratio of gene expression values determined in *V. cholerae tcpPH* mutant compared to N16961 strain; column (AL): log_2_ ratio of gene expression values determined in *V. cholerae toxT* mutant compared to N16961 strains. Click here for file

Additional file 11 **Table S7. **Analysis of the transcription start sites and corrections introduced. Column (A): locus tag; column (B): position of the start codon identified in our analysis; column (C): position of the TSS identified in the previous gene finding performed using Glimmer and Orpheus software; column (D): modification introduced; column (E): strand; columns (F-G): sequence and score of the ribosome binding site identified using MotifScanner software (the RBS site and the start codon are labeled in red); column (H): gene description; column (I): gene finding software (if any) corroborating the new start codon identified; column (J): description of the alignment performed using BLAST, alignments that agree with our start codon prediction have been reported; column (K): agreement with the structure of the transcript identified using RNA-seq, genes not confirmed using this method were modified considering only bioinformatics (RBS position and BLAST with other bacteria); column (M) sequence determined considering the new gene prediction. Click here for file

Additional file 12 **Table S8. **sRNAs identified using RNA-seq are classified considering their position with respect to protein coding genes. Column (A): locus tag was arbitrarily assigned considering the locus tag of the gene overlapped or the closer gene and adding “a”, “b” or “c” to the locus tag; column (B): chromosome; columns (C-D): transcript start and transcript end determined considering Rfam database and/or RNA-seq data; column (E): strand; column (F): gene name; column (G): comment; column (H): genes localized in repeated regions are labeled; column (I): protein-coding genes overlapped to the sRNA; column (J): sRNAs overlapped to ribosome binding sites of protein-coding genes are reported. Click here for file

Additional file 13 **Figure S4. **Minimum free energy [kcal/mol] determined using RNAfold software (Vienna package). Data refer to the 5^′^-UTR regions (A) and to different classes of sRNAs: intergenic (B), partially overlapped (C) and completely overlapped (D) to the ORFs. Grey points correspond to random sequences having base composition equivalent to that of the RNAs reported in the same analysis. Blue and red lines represent lowess interpolation of random sequences and putative small RNAs. Click here for file

Additional file 14 **Table S9. **Analyses of the 5^′^ and 3^′^-UTRs length for genes belonging to different COG classes. We reported the minimum (columns E, O), the first quartile (columns F, P), the median (columns G, Q), the mean (columns H, R) the third quartile (columns I, S) and the maximum length (columns J, T) of UTRs for genes classified considering COG classes (columns D, N). Genes having UTRs significantly longer (black bold) or shorter (red bold) are highlighted. We have considered only classes having p-value lower than 5% (Wilcoxon test). Results are reported in graph in Figure 5. Click here for file

Additional file 15 **Table S10. **Length of the 5^′^ and 3^′^-UTRs determined using RNA-seq. Column (A): reports if 5^′^-UTR or 3^′^-UTR length was determined for a specific transcript; columns (B,C): start/end determined for each UTR region; column (D): strand of the gene (or operon) analyzed; columns (E-L): gene(s) belonging to the transcript; columns (M-T) - COG categories of the genes belonging to the transcript. column (U): UTR length. Click here for file

Additional file 16 **Figure S5. **Histogram reporting 1153 distance values between the transcription termination sites determined using RNA-seq and the closest rho-independent terminator. Positive values indicate that termination site determined with RNA-seq is upstream of the predicted rho-independent terminator. The size of the 3^′^-UTRs is slightly underestimated in RNA-seq, in fact the distribution is centered on positive values (28 bp) and the size of the 3^′^-UTRs determined considering the terminator is generally larger than that determined with RNA-seq. Click here for file

Additional file 17 **Figure S6. **Venn diagrams showing the number of differentially expressed genes identified in the comparisons: Since the number of possible intersections between subgroups is very high, to obtain a better visualization, data are subdivided into four diagrams. In (A) it is evident that blue (genes down regulated in DB110 LP *vs.* DB110 HP comparison) and yellow (genes down regulated in TW30 LP *vs.* TW30 HP comparison) ovals have only limited overlap and this indicates that genes differentially expressed in parental and mutant strains at different pressures are only partially coincident. The same is true for genes up-regulated in “DB110 LP *vs.* DB110 HP” and “TW30 LP *vs.* TW30 HP” comparisons, reported in (B), blue and yellow ovals. In (A) the arrow indicates the genes that have an expression similar to *ompH*, this number is higher if compared to those having a “*ompL* like” behaviour (reported in D). Click here for file

Additional file 18 **Table S13. **Analysis of the COG classes enriched in differentially expressed gene performed using hypergeometric distribution. In the upper part of the table (rows 1–22) analyses was performed considering only the more significant differentially expressed genes (p-value <= 0.0001; log_2_ ratio >= 1 or log_2_ ratio <= −1), in the lower part of the figure (rows 24–45) analysis was performed considering all genes differentially expressed (p-value <= 0.001; log_2_ ratio >= 0.7 or log_2_ ratio <= −0.7). column (A): COG class; column (B): total number of genes belonging to each COG class in chrs 1–2 and in plasmid; columns (C, F, H, J): number of differentially expressed genes identified in each comparison; columns (D, G, I, K): p-values calculated for each comparison; column (E): total number of genes belonging to each COG class in chr. 1 and in chr. 2; column (L): COG description. Click here for file

Additional file 19 **Table S12. **Gene Ontology analysis performed considering differentially expressed genes. Analyses were performed using GoMiner software and considering only the more statistically significant data (p-value <= 0.0001; log_2_ ratio >= 1 or log_2_ ratio <= −1). Genes up and down-regulated on each comparison are considered separately. In column (A) is reported the description of the GO classes identified. For each comparison are reported the total number of genes belonging to each GO biological process class (columns B, H, N, T, Z, AF, AL, AR), the number of genes up or down-regulated (columns C, I, O, U, AA, AG, AM, AS), the enrichment value calculated by the software (columns D, J, P, V, AB, AH, AN, AT) and the p-value (E, K, Q, W, AC, AI, AO, AU). Click here for file

Additional file 20 **Table S14. **Putative ToxR regulated genes. Column (A): locus tag; column (B): Swiss-prot ID; column (C): gene name; column (D): gene description downloaded from NCBI database; column (E): gene description obtained from CMR (Comprehensive Microbial Resource) (http://cmr.jcvi.org/tigr-scripts/CMR/CmrHomePage.cgi); columns (F-G): COG code and COG ID; columns (H, I, J, K): chromosome, strand, start and stop position of the genes; columns (L, M, N, O): number of reads mapped on each gene in the four experiments (DB110 at 28 MPa, DB110 at 0.1 MPa, TW30 at 28 MPa and TW30 at 0.1 MPa). All values are normalized considering the total number of reads mapped on the reference sample (DB110 at 28 MPa); columns (P, Q): log_2_ ratios and p-values calculated using DEGseq software for the comparison between DB110 at 0.1 MPa and DB110 at 28 MPa; columns (R, S): log_2_ ratios and p-values calculated using DEGseq software for the comparison between TW30 at 28 MPa and DB110 at 28 MPa; columns (T, U): log_2_ ratios and p-values calculated using DEGseq software for the comparison between TW30 at 0.1 MPa and DB110 at 0.1 MPa; columns (V, W): log_2_ ratios and p-values calculated using DEGseq software for the comparison between TW30 at 0.1 MPa and TW30 at 28 MPa; column (X): distance between transcription profile of each gene and that of *ompH*, calculated considering the Pearson correlation. Click here for file
