# Supplementary figures and images for: The transcriptional landscape of the deep-sea bacterium Photobacterium profundum in both a toxR mutant and its parental strain
Source: BMC Genomics. 2012 Oct 29;13:567. doi: 10.1186/1471-2164-13-567 (PMC3505737; doi:10.1186/1471-2164-13-567)

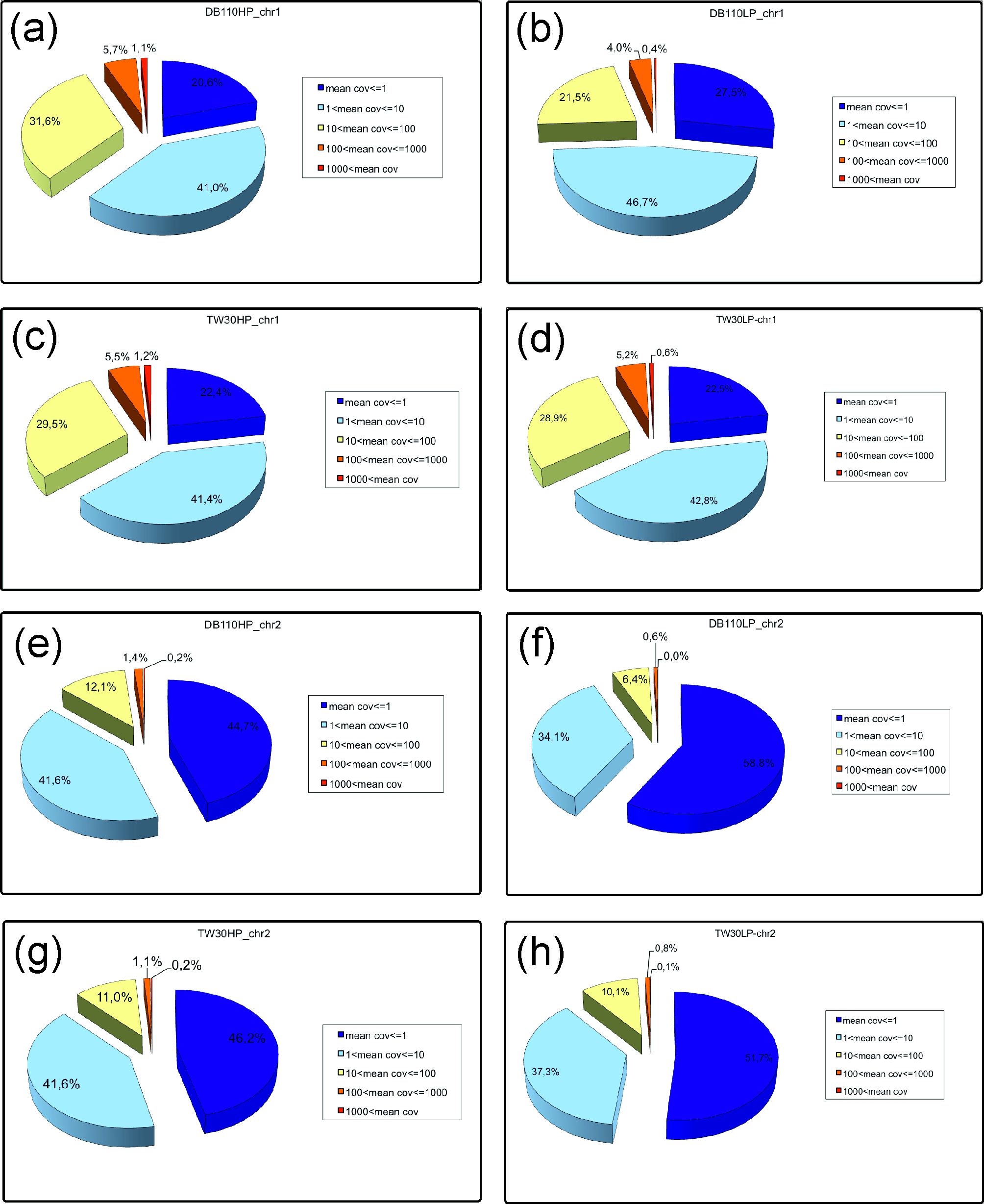

Supplement: Additional file 2 — Figure S1. Pie charts reporting the coverage of the P. profundum SS9 genes. (A-D) coverage of the chr. 1 genes, (E-H) coverage of the chr. 2 genes. Coverage was calculated at a single base level on the genome considering the uniquely aligned SOLiD reads and then converted to the mean coverage value for each gene. [file 1471-2164-13-567-S2.tiff]

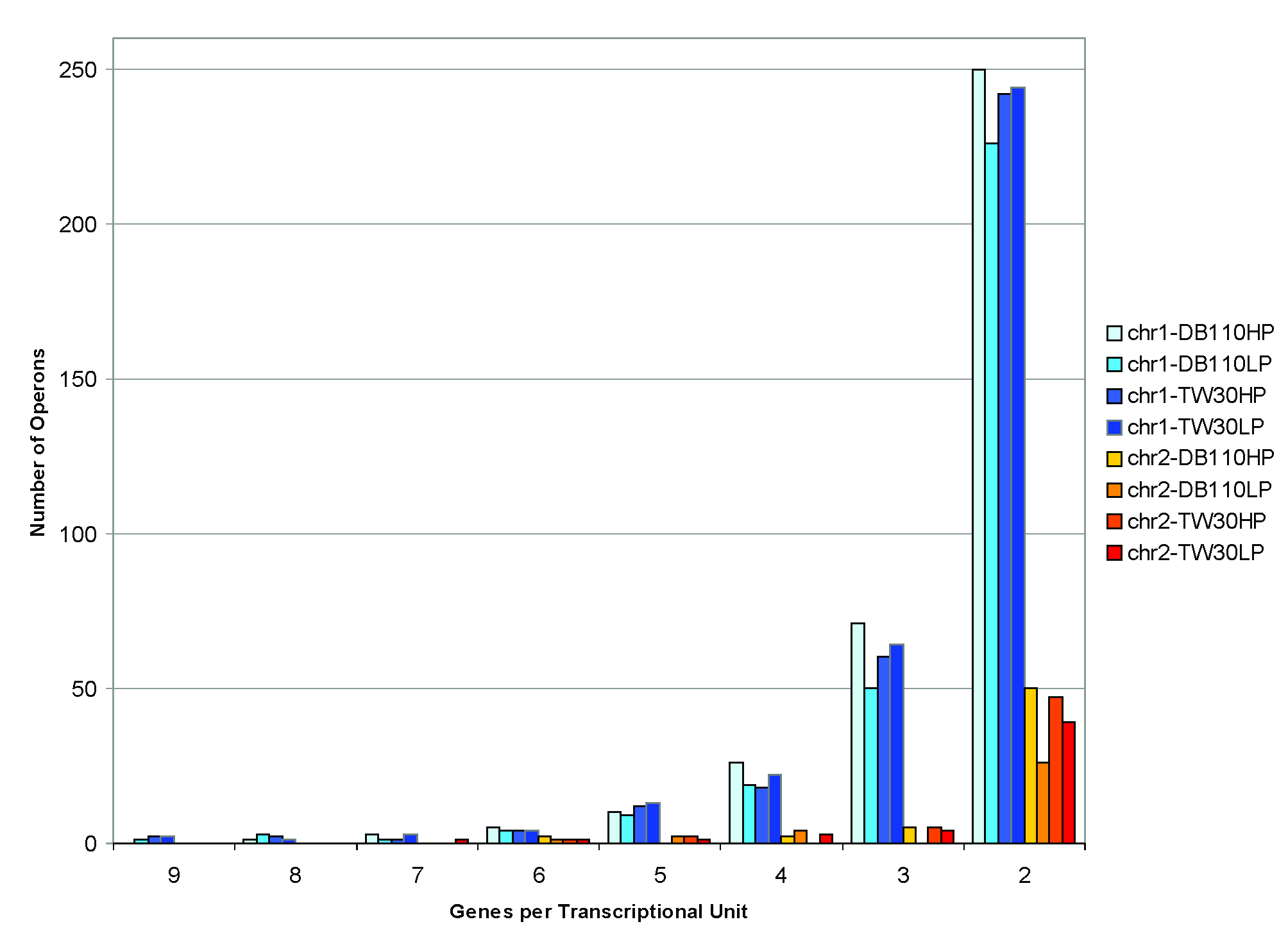

Supplement: Additional file 5 Figure S2. — Number of genes transcribed in operons of different lengths in DB110 and TW30 strains at low and high pressure. From the histogram it is clearly evident that operons composed by small number of genes are more abundant. This is a general trend in Bacteria. Data were reported for chr. 1 (azure-blue) and chr. 2 (red-orange) and the result is similar. chr. 2 has on average a lower percentage of genes organized in operons but in this graph the result is also due to the absolute gene number that is lower on chr. 2 than on chr. 1. [file 1471-2164-13-567-S5.tiff]

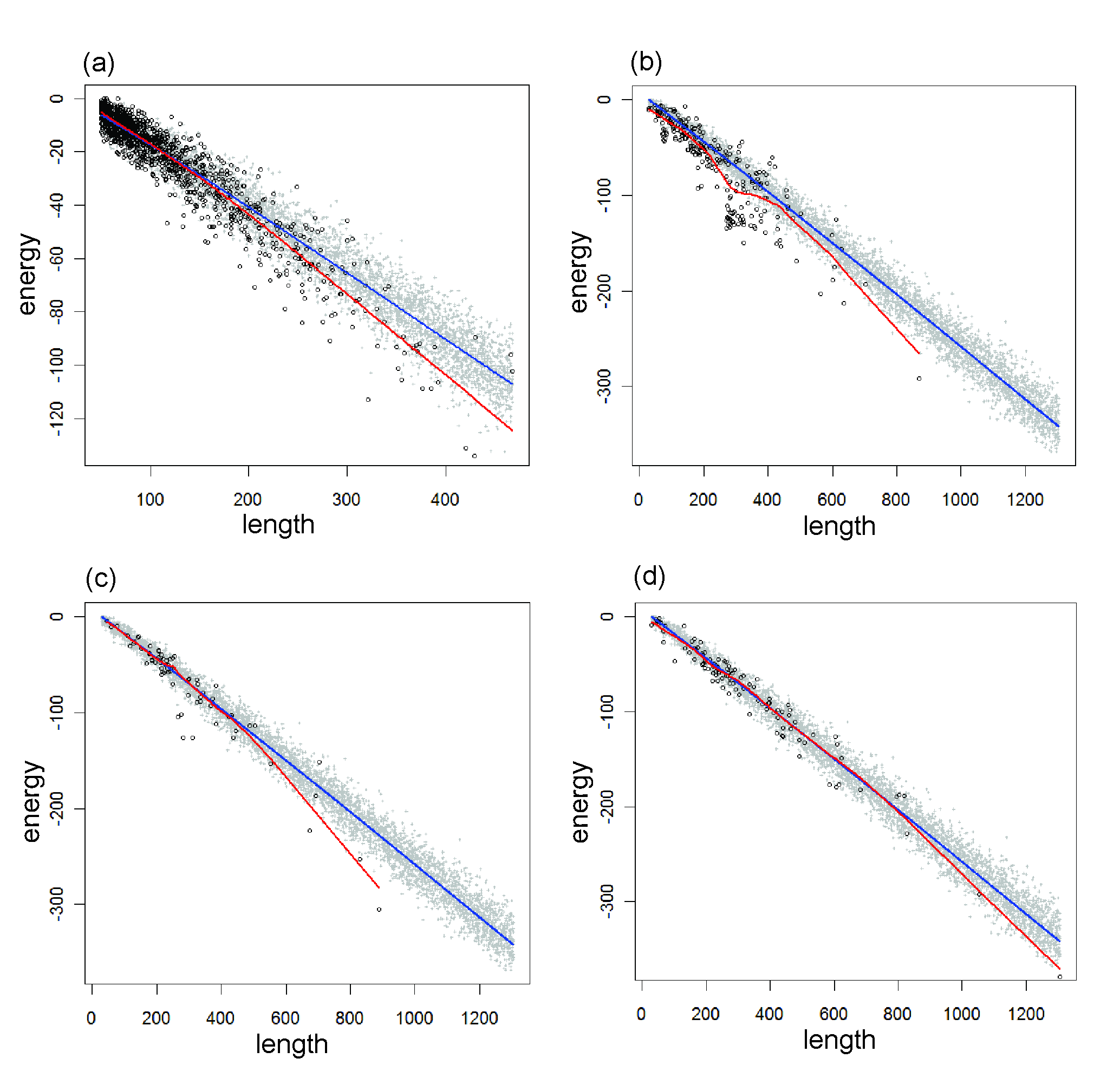

Supplement: Additional file 13 — Figure S4. Minimum free energy [kcal/mol] determined using RNAfold software (Vienna package). Data refer to the 5′-UTR regions (A) and to different classes of sRNAs: intergenic (B), partially overlapped (C) and completely overlapped (D) to the ORFs. Grey points correspond to random sequences having base composition equivalent to that of the RNAs reported in the same analysis. Blue and red lines represent lowess interpolation of random sequences and putative small RNAs. [file 1471-2164-13-567-S13.tiff]

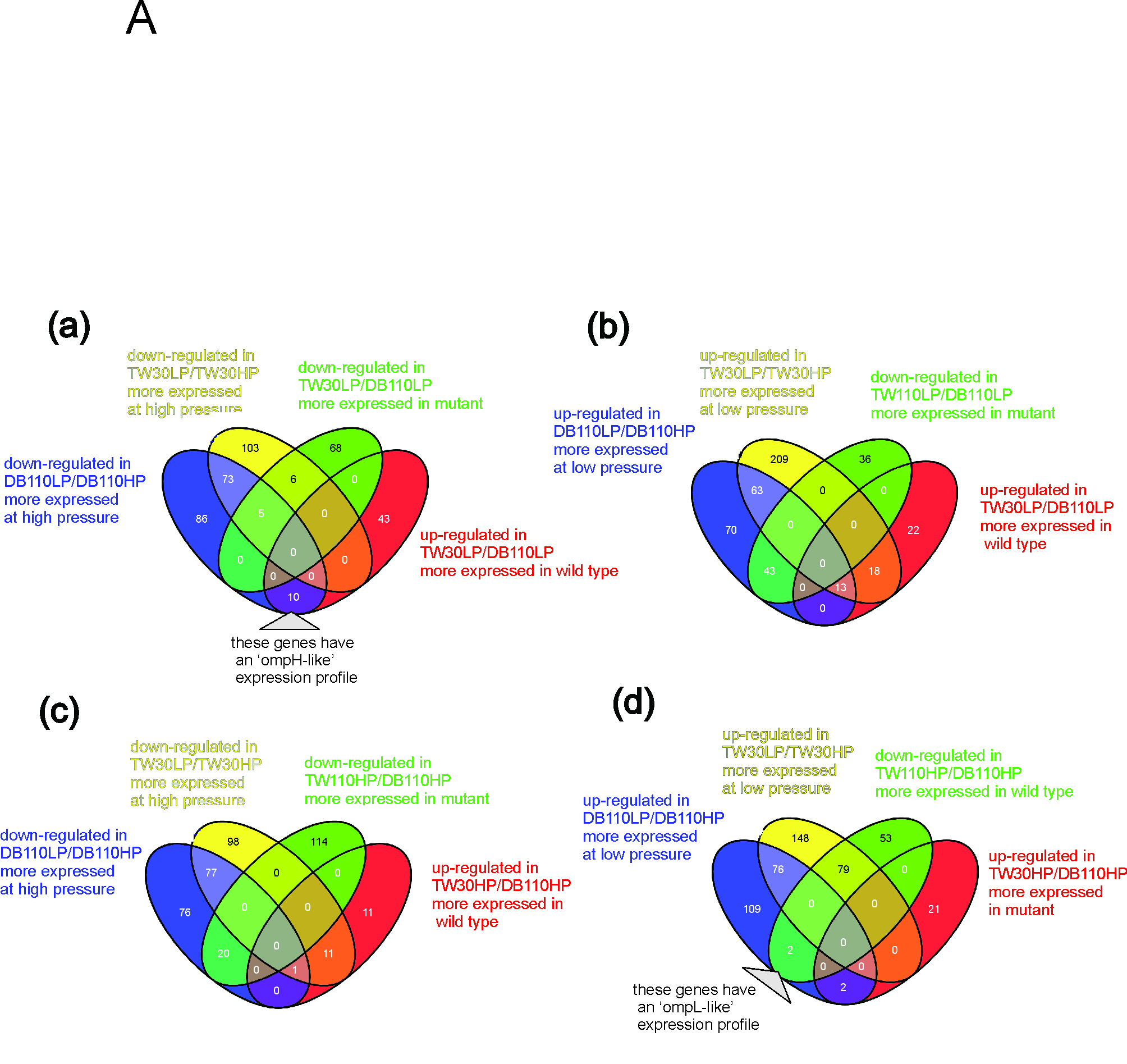

Supplement: Additional file 17 — Figure S6. Venn diagrams showing the number of differentially expressed genes identified in the comparisons: Since the number of possible intersections between subgroups is very high, to obtain a better visualization, data are subdivided into four diagrams. In (A) it is evident that blue (genes down regulated in DB110 LP vs. DB110 HP comparison) and yellow (genes down regulated in TW30 LP vs. TW30 HP comparison) ovals have only limited overlap and this indicates that genes differentially expressed in parental and mutant strains at different pressures are only partially coincident. The same is true for genes up-regulated in “DB110 LP vs. DB110 HP” and “TW30 LP vs. TW30 HP” comparisons, reported in (B), blue and yellow ovals. In (A) the arrow indicates the genes that have an expression similar to ompH, this number is higher if compared to those having a “ompL like” behaviour (reported in D). [file 1471-2164-13-567-S17.tiff]
